# Hydrogen Production by Steam Reforming of Acetone–Butanol–Ethanol over Supported Nickel Catalysts: Effect of MgO Promoter

**DOI:** 10.1021/acsomega.5c06102

**Published:** 2025-09-09

**Authors:** Arthur Rieger Q. Gonçalves, João Paulo da S. Q. Menezes, Robinson L. Manfro, Mariana M. V. M. Souza

**Affiliations:** † 28125 Escola de Química − Universidade Federal do Rio de Janeiro (UFRJ), Centro de Tecnologia, Bloco E, sala 206, 21941-909 Rio de Janeiro, RJ, Brazil; ‡ Navy Research Institute (IPqM), 21931-095 Rio de Janeiro, RJ, Brazil

## Abstract

Hydrogen is rapidly emerging as a cornerstone of sustainable energy strategies, and unlocking its full potential depends on innovative technologies that harness renewable raw materials. In this context, this study explores a promising route: hydrogen production using an ABE (acetone–butanol–ethanol) mixture derived from the fermentation of various biomass substrates. A Ni/MgO-Al_2_O_3_ catalyst (20 wt % of NiO and 10 wt % of MgO) was prepared and compared with Ni/Al_2_O_3_ in ABE (3:6:1 mass ratio, 10% v/v) steam reforming at 400–600 °C. The MgO-promoted catalyst demonstrated significantly enhanced activity due to its superior Ni dispersion and increased basicity. At 500 °C, the Ni/MgO-Al_2_O_3_ catalyst achieved 92% global ABE conversion and a hydrogen yield of 55% while maintaining stable performance over 30 h on stream. Moreover, the coking rate was substantially reduced compared with that of Ni/Al_2_O_3_, and the deposited carbon was less amorphous, indicating an improved resistance to deactivation. These findings highlight Ni/MgO-Al_2_O_3_ as a promising catalyst for sustainable hydrogen production from biomass-derived ABE mixtures.

## Graphical abstract

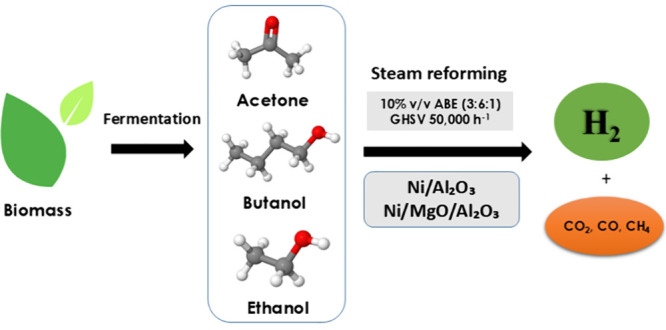

## 1 Introduction

The widespread use of fossil fuels has significantly increased greenhouse gas emissions, leading to global warming. Concerns about global warming are rising due to scientific evidence, public awareness, and the visible impacts of climate change. Hydrogen is a key contributor to more sustainable development because it has the potential to be a clean energy carrier, particularly in sectors that are challenging to electrify. In 2022, global demand for hydrogen reached 95 Mt and is projected to increase to 430 Mt per year by 2050 under a Net Zero Emissions (NZE) Scenario.^1,2^

Hydrogen production today is mainly based on fossil fuels, especially natural gas. Only 2% comes from renewable sources, mostly through water electrolysis, with biomass playing no significant role at an industrial scale.^3^ Biomass can be considered a carbon-neutral hydrogen source because the CO_2_ released during the reforming or gasification process is roughly equivalent to the CO_2_ absorbed by the plants through photosynthesis during their growth.

The biomass materials available as feedstock for hydrogen production can be categorized into four types: lignocellulosic biomass, agricultural residues, biowastes, and energy crops.^4^ The production of lignocellulosic biomass was reported to be about 4.6 and 7 billion tons annually from agriculture and forest residues, respectively. Thus, the potential application of lignocellulosic biomass for hydrogen production has to be considered.^3,5^

Acetone–butanol–ethanol (ABE) mixture is produced by the fermentation of biomass-derived substrates, such as sugars, starch, and lignocellulosic materials. The utilization of starch and sugars as substrates represents the main production costs of ABE and falls under the food vs fuel category of the first-generation feedstocks.^6^ Lignocellulosic biomass is an abundant and low-cost feedstock that does not compete with human food; thus, it is proposed as an alternative source for commercial ABE production. However, the pretreatment and hydrolysis process required to convert lignocellulosic materials into sugars can significantly increase the capital and operating costs.^6,7^ ABE yield from lignocellulosic materials can be improved through pretreatment techniques and/or metabolic engineering of the fermenting strains.^7^ The acetone, butanol, and ethanol from ABE fermentation are typically produced at a mass ratio of 3:6:1, respectively.^7,8^

Although acetone, butanol, and ethanol have been extensively used separately for hydrogen production, very few research papers are available in the literature on reforming the ABE mixture.^9–11^ Reforming the raw mixture is favorable in terms of energy, as no further separation process is necessary. The most common ABE separation technique is distillation, which has high investment costs and low selectivity.^8^

Cai et al.^9,10^ evaluated Co-based catalysts for the steam reforming of ABE mixture. In their first study, cobalt catalysts supported on ZnO were tested in steam reforming at 600 °C for 90 h on stream, showing that the addition of Ir to Co/ZnO inhibited coke formation and increased H_2_ concentration.^9^ Bimetallic Co–Ir catalysts supported on Ce_0.82_Zr_0.18_O_2_ were evaluated in another study^10^ for oxidative steam reforming of ABE mixture: the calcination temperature of the support influenced its basicity and oxygen storage capacity, which was correlated to the catalyst activity. Wang et al.^11^ investigated the effect of metal particle size of Ni catalysts supported on mesoporous MgO for butanol and ABE steam reforming (at 450 °C and 6 h on stream). The maximum H_2_ yield was obtained with Ni particles of about 6 nm for both reactions, but the catalyst with the smallest Ni particle size (about 3.6 nm) performed much worse in ABE conversion than in butanol. Thus, it was concluded that multimolecule activation in ABE reforming requires Ni ensembles large enough to accommodate multiple bond cleavage reactions.

Nickel catalysts are widely used in the reforming of oxygenated compounds due to their high activity in breaking C–C, O–H, and C–H bonds and their low cost. However, they are particularly prone to deactivation by coke deposition.^12–15^ The selection of support is crucial for catalyst properties, influencing active phase dispersion and resistance to carbon formation. Alumina is one of the most employed supports for reforming catalysts, thanks to its high specific surface area, enabling a good metallic phase dispersion. However, the acidic sites on alumina can promote coke deposition through dehydration, cracking, and polymerization reactions.^16,17^ Adding basic promoters like MgO to the alumina support is a promising strategy to reduce acidity and minimize carbon formation.

Sánchez-Sánchez et al.^18^ showed that Ni/MgO-Al_2_O_3_ exhibited higher catalytic activity for ethanol steam reforming in comparison to Ni/Al_2_O_3_ because MgO decreased the support acidity and modified the Ni–Al_2_O_3_ interaction, which might improve the Ni dispersion. Similar results were reported by Navarro et al.^19,20^ for acetone steam reforming, showing that MgO decreases the production of coke and oligomers on Ni catalysts. We recently reported that the Ni/MgO/Al_2_O_3_ catalyst presented an outstanding performance in butanol steam reforming regarding butanol conversion, hydrogen yield, and time-on-stream stability.^21^

This study aims to assess the role of MgO as a basic promoter in nickel-supported alumina catalysts for the ABE reforming reaction. Additionally, it explores the correlation between catalyst properties and performance while examining the relationship between catalyst stability and coke deposition. The novelty of this study lies in investigating this catalyst for ABE reforming and evaluating the effect of the competitive conversion of the three components on the hydrogen yield and resistance to coke formation.

## 2 Experimental Section

### 2.1 Catalyst Preparation and Characterization

The nickel catalyst supported on MgO-Al_2_O_3_ (denoted as NiMgAl) was synthesized by wet impregnation, with theoretical NiO and MgO loadings of 20 and 10 wt %, respectively. The support of pure γ-alumina was prepared by calcination of a commercial boehmite (Sasol) at 500 °C for 3 h using a 60 mL min^–1^ air flow. To prepare MgO-Al_2_O_3_, a measured amount of magnesium nitrate (Mg­(NO_3_)_2_·6H_2_O) (Sigma-Aldrich) was dissolved in deionized water and added to γ-Al_2_O_3_ using a rotary evaporator. The mixture was rotated at room temperature for 2 h to ensure homogenization, followed by water removal through evaporation at 80 °C under vacuum. The resulting material was then dried at 100 °C for 24 h and calcined at 500 °C for 3 h under flowing air (60 mL min^–1^). The catalyst was prepared by impregnation of nickel nitrate (Ni­(NO_3_)_2_·6H_2_O) (Sigma-Aldrich) on the MgO-Al_2_O_3_ support, using the same steps. A reference catalyst without MgO (denoted as NiAl) was also synthesized using the same methodology.

The prepared catalysts were characterized by X-ray fluorescence (XRF), X-ray diffraction (XRD), N_2_ physisorption, temperature-programmed reduction (TPR), and temperature-programmed desorption of NH_3_ (NH_3_-TPD) and CO_2_ (CO_2_-TPD), as presented in a previous paper.^21^ Some of these characterizations are summarized in Table 1. The theoretical and actual NiO and MgO contents are very close.

**Table 1:** Chemical Composition, Textural Properties, Ni Crystallite Size, Ni Dispersion, Amount of Desorbed NH_3_ from TPD-NH_3_, and Amount of Desorbed CO_2_ from TPD-CO_2_ ^21^

Catalyst	NiO (wt %)	MgO (wt %)	BET area (m^2^ g^–1^)	Pore volume (cm^3^ g^–1^)	Pore size (Å)	Ni crystallite size (nm)^a^	Ni dispersion (%)^a^	μmol NH_3_ g^–1^	μmol CO_2_ g^–1^
NiAl	21	-	145	0.34	65.4	8.4 ± 2.0	12.0	458.9	76.5
NiMgAl	22	9	66	0.07	72.3	5.6 ± 1.6	18.0	71.5	182.3

a After reduction at 800 °C.

### 2.2 Catalytic Tests

The reforming reactions were performed in a fixed-bed reactor of quartz at atmospheric pressure. Each test was carried out with 150 mg of the catalyst mixed with 750 mg of silicon carbide, which were reduced *in situ* under 33% H_2_/N_2_ flow (90 mL min^–1^) up to 800 °C at a heating rate of 10 °C min^–1^, maintaining this temperature for 1 h. The temperature of reduction was chosen based on the reduction profiles (TPR) of the catalysts.^20^

The reactor was fed with a 10% v/v ABE aqueous solution (mass ratio of acetone–butanol–ethanol of 3:6:1, respectively) at 0.259 mL min^–1^ through an Eldex 1SM pump. The vaporization of this solution was carried out at 200 °C under He flow (127.6 mL min^–1^), which was calculated to represent 20% v/v of the total gas flow. DWSIM software was employed to determine the total inlet gas flow and pump flow, using a Gibbs reactor and Peng–Robinson package, aiming at a gas hourly space velocity (GHSV) of 50,000 h^–1^. The lines through which the vaporized reagents passed were also kept at the same temperature to prevent condensation. The effect of the temperature was evaluated at 400, 500, and 600 °C, with each temperature maintained for 2 h. Stability tests for up to 30 h were performed at 500 °C.

The products from the reactor passed through a condenser at 4 °C, resulting in the separation of the liquid and gaseous phases. The liquid phase was collected and analyzed by a high-performance liquid chromatograph (HPLC) (Shimadzu Prominence) equipped with a Bio-Rad Aminex HPX-87H column (300 × 7.8 mm), using 0.005 M H_2_SO_4_ as the eluent at 0.6 mL min^–1^, and both UV and refractive index detectors. The column was kept at 30 °C, and each run lasted 40 min.

The gas phase was analyzed online by a Shimadzu GC-2014 gas chromatograph (GC), using He as a carrier gas with a column flow of 2.78 mL min^–1^. The GC was equipped with a Carboxen 1010 column, coupled to a thermal conductivity detector (TCD), for the analysis of H_2_, CO, CO_2_, and CH_4_, and an RT-QPLOT column, connected to a flame ionization detector (FID), for the analysis of hydrocarbons. The columns were kept at 40 °C for 11 min and then heated at 15 °C min^–1^ up to 180 °C for 5 min.

The catalyst performance was evaluated according to the equations described below.

Conversion of each component:

1 $$X_{i}\left(\%\right)=\frac{N_{i}^{In}-N_{i}^{Out}}{N_{i}^{In}}\times 100$$

where *N*_*i*_^*In*^ is the molar flow rate of component *i* in the feed, and *N*_*i*_^*Out*^ is the molar flow rate of component *i* after reaction (*i* = acetone, butanol, or ethanol).

Global conversion:

2 $$X\left(\%\right)=\sum X_{i}\left(\%\right)\times F_{i}$$

where *F_i_* is the molar fraction of each component in the feed.

Conversion to gas:

3 $$X_{G}\left(\%\right)=\frac{\mathrm{carbon molar flow rate in gas phase}}{\mathrm{carbon molar flow rate in feed}}\times 100$$

H_2_ yield:

4 $$Y_{H_{2}}\left(\%\right)=\frac{N_{H_{2}}^{Out}}{8N_{Acetone}^{In}+12N_{Bu\tan ol}^{In}+6N_{Ethanol}^{In}}\times 100$$

where *N*_*H*_2__^*Out*^ is the molar flow rate of hydrogen produced.

CO_2_, CO, CH_4_, and C_2_H_4_ selectivity:

5 $$Y_{j}\left(\%\right)=\frac{N_{j}^{Out}}{\sum N_{j}^{Out}}\times 100$$

where *N*_*j*_^*Out*^ is the carbon molar flow rate of each product (*j* = CO_2_, CO, CH_4_, or C_2_H_4_).

### 2.3 Characterization of Catalysts Used in Stability Tests

X-ray diffraction (XRD) measurements were carried out in a Rigaku Miniflex II diffractometer, equipped with a graphite monochromator, using Cu Kα radiation operating at 30 kV and 15 mA. The analyses were performed in the range of 5° ≤ 2θ ≤ 90° with steps of 0.05° and a counting time of 1 s for each step. The spent catalysts were analyzed without any treatment after the reaction.

Thermogravimetric analysis (TGA) and differential thermal analysis (DTA) were performed using a TA SDT Q600 instrument to quantify coke deposition in the spent catalysts. Samples containing approximately 10 mg were heated to 1000 °C at a rate of 10 °C min^–1^ under a synthetic air flow of 100 mL min^–1^.

The coke structure of the spent catalysts was investigated by Raman spectroscopy using an Xplora Plus Horiba DXR Raman Microscope equipped with a CCD detector and a 10× objective lens. The samples were excited by a 638 nm laser at 1000–2000 cm^–1^. The spectrum acquisition was performed with 10 scans and an exposure time of 10 s. Three different spectra were acquired in various sample spots, and the intensity ratio was calculated as the arithmetic mean of the spectra.

The morphology of coke formed on catalysts was also evaluated by scanning electron microscopy (SEM) using a Hitachi TM-3030 microscope. The voltage acceleration employed was 15 kV with secondary and backscattering electrons.

## 3 Results and Discussion

### 3.1 Catalytic Tests

The effect of the temperature on the steam reforming of the ABE mixture was evaluated at 400, 500, and 600 °C, each with a catalyst sample, during 2 h of reaction. Figure 1A shows the acetone, butanol, and ethanol conversion as well as global conversion, and Figure 1B shows the H_2_ yield and conversion to gas at each temperature. At 400 °C, butanol showed higher conversion than acetone and ethanol, and at 500 °C the opposite occurred. Thus, the increase in temperature favors the conversion of lighter oxygenates. The global conversion was 87.5% for NiMgAl and 81.9% for NiAl at 500 °C. The conversion was 100% for the three components at 600 °C in both catalysts. This behavior suggests that butanol is preferentially adsorbed at low temperatures, hindering the access of acetone and ethanol to Ni active sites. When the temperature increases, acetone and ethanol compete more severely for the active sites, reducing butanol conversion.

**Figure 1:**
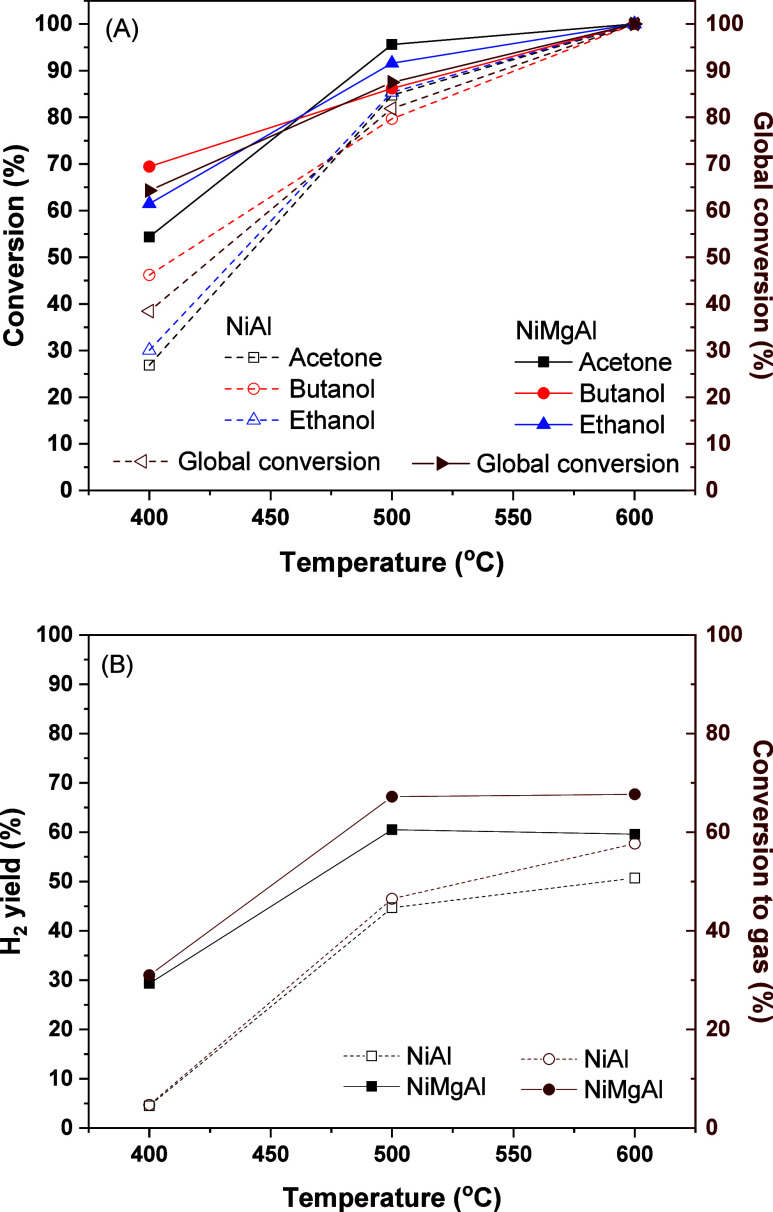
Effect of temperature on (A) acetone, butanol, and ethanol conversion and global conversion. (B) Effect of temperature on H_2_ yield and conversion to gas. Reaction conditions: 10% v/v ABE solution, GHSV = 50,000 h^–1^.

The NiMgAl catalyst presented higher conversions to gas and H_2_ yields than NiAl. The conversion to gas was 67.2% for NiMgAl and 46.5% for NiAl at 500 °C. Since no byproducts were identified in the liquid phase, the difference between the global conversion and the conversion to gas mostly represents the formation of coke or other unidentified carbon products. Concerning H_2_ yield, it was 60.5% for NiMgAl and 44.7% for NiAl at 500 °C.

The higher values for global conversion, conversion to gas, and H_2_ yield presented by the NiMgAl catalyst at all temperatures indicate its higher activity in the steam reforming reaction. The highest conversions presented by the NiMgAl catalyst may be related to its higher metallic dispersion and basicity (Table 1), as observed for butanol steam reforming.^21^ It is known that basic sites promote water dissociation and the subsequent formation of OH groups, which can react with carbon species on metal sites, enhancing hydrogen formation.^18,21–24^ Wang et al.^11^ obtained a maximum of 95% butanol conversion and 70% H_2_ yield using a Ni/MgO catalyst with a Ni particle size of 6 nm in ABE reforming at 450 °C (6 h of reaction). The conversion of the other components was not reported. These values are comparable to those obtained with the NiMgAl catalyst at 500 °C, considering the differences in reaction conditions (0.3 g of catalyst, N_2_ flow of 45 mL min^–1^, and liquid flow of 0.092 mL min^–1^).

The selectivity for carbon-containing gas products is shown in Figure 2. CO_2_ is the main carbon product, as expected by the reforming reactions (eqs 6–8). CO_2_ selectivity increases with temperature from 400 to 500 °C, while the CO selectivity decreases, due to the favoring of endothermic reforming reactions over the water–gas shift (eq 9). The formation of CH_4_ at low temperatures is associated with methanation reactions (eqs 10 and 11). The small increase in the CO selectivity from 500 to 600 °C can be attributed to methane steam reforming (reverse of eq 10), which is highly endothermic.

6 $$C_{4}H_{10}O+7H_{2}O⇌4{\mathrm{CO}}_{2}+12H_{2}$$

7 $$C_{3}H_{6}O+5H_{2}O⇌3{\mathrm{CO}}_{2}+8H_{2}$$

8 $$C_{2}H_{6}O+3H_{2}O⇌2{\mathrm{CO}}_{2}+6H_{2}$$

9 $$\mathrm{CO}+H_{2}O⇌{\mathrm{CO}}_{2}+H_{2}$$

10 $$\mathrm{CO}+3H_{2}⇌{\mathrm{CH}}_{4}+H_{2}O$$

11 $${\mathrm{CO}}_{2}+4H_{2}⇌{\mathrm{CH}}_{4}+2H_{2}O$$

**Figure 2:**
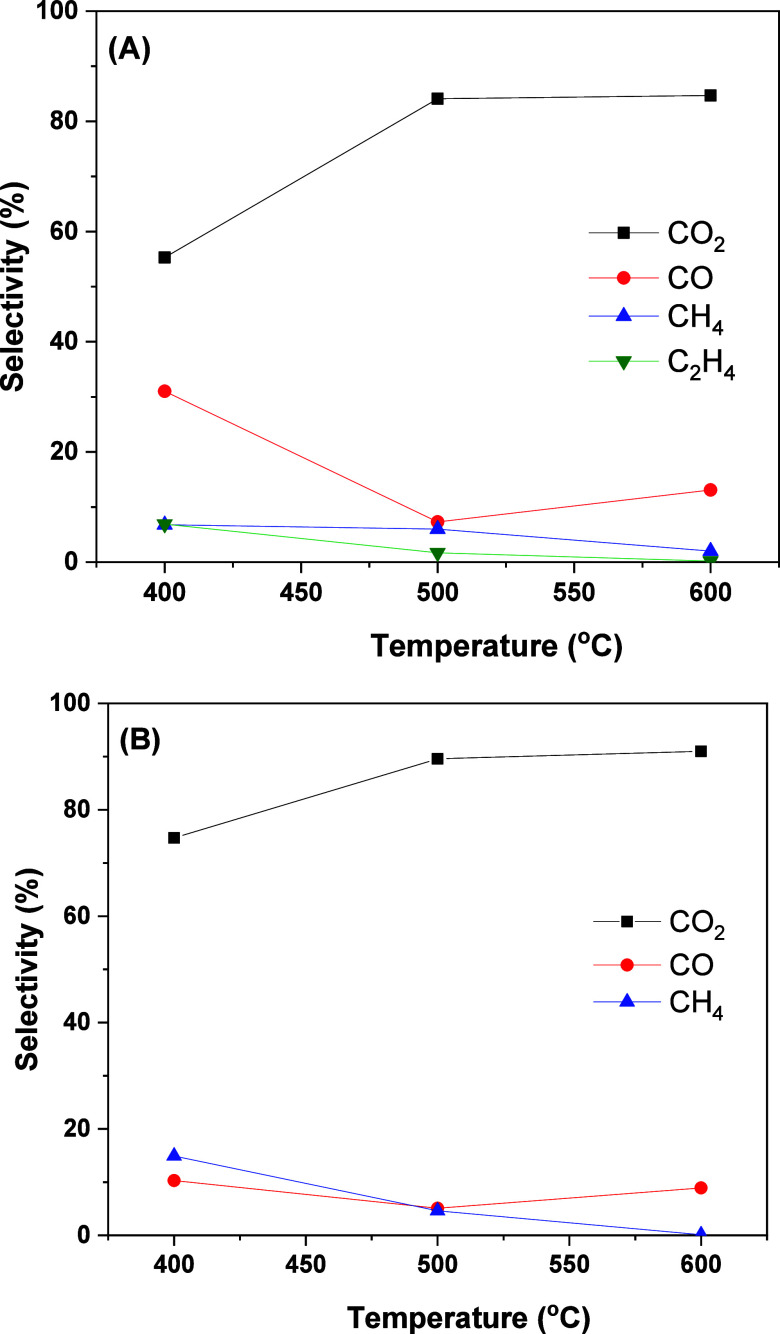
Effect of temperature on selectivity to gaseous products containing carbon for (A) NiAl and (B) NiMgAl catalysts. Reaction conditions: 10% v/v ABE solution, GHSV = 50,000 h^–1^.

The NiAl catalyst showed ethylene formation, which can be mainly related to ethanol dehydration (eq 12). The introduction of MgO suppressed ethylene formation because dehydration is favored on acid sites.^12–14,18,25^ Ethylene can also be formed from ketene, produced by acetone decomposition (eqs 13 and 14).^26^

12 $$C_{2}H_{6}O⇌C_{2}H_{4}+H_{2}O$$

13 $$C_{3}H_{6}O⇌{\mathrm{CH}}_{2}\mathrm{CO}+{\mathrm{CH}}_{4}$$

14 $$2{\mathrm{CH}}_{2}\mathrm{CO}⇌2\mathrm{CO}+C_{2}H_{4}$$

The temperature of 500 °C was chosen to evaluate the stability of the catalysts after 30 h of reaction because the conversion and H_2_ yield almost did not increase from this temperature. The global conversion, conversion to gas, and H_2_ yield obtained in the stability tests are shown in Figure 3. The NiMgAl catalyst showed the highest conversions and the greatest stability, with a global conversion of about 92% and H_2_ yield of 55%, over the 30 h of reaction, without any apparent deactivation. On the other hand, the NiAl catalyst presented a global conversion of approximately 82% in the first 3 h of reaction; in the fourth hour, there was an abrupt drop to 43%. Before completing the fifth hour, the catalytic system already presented pressure in the line, and no gases exited the reactor. The reactor was completely blocked after this period, which was related to excessive coke formation, as will be shown later. The deactivation degree, calculated by a linear fit of the global conversion vs time on stream (TOS), is displayed in Table 2. The deactivation degree was null for NiMgAl and very high (9.7% h^–1^) for NiAl.

**Figure 3:**
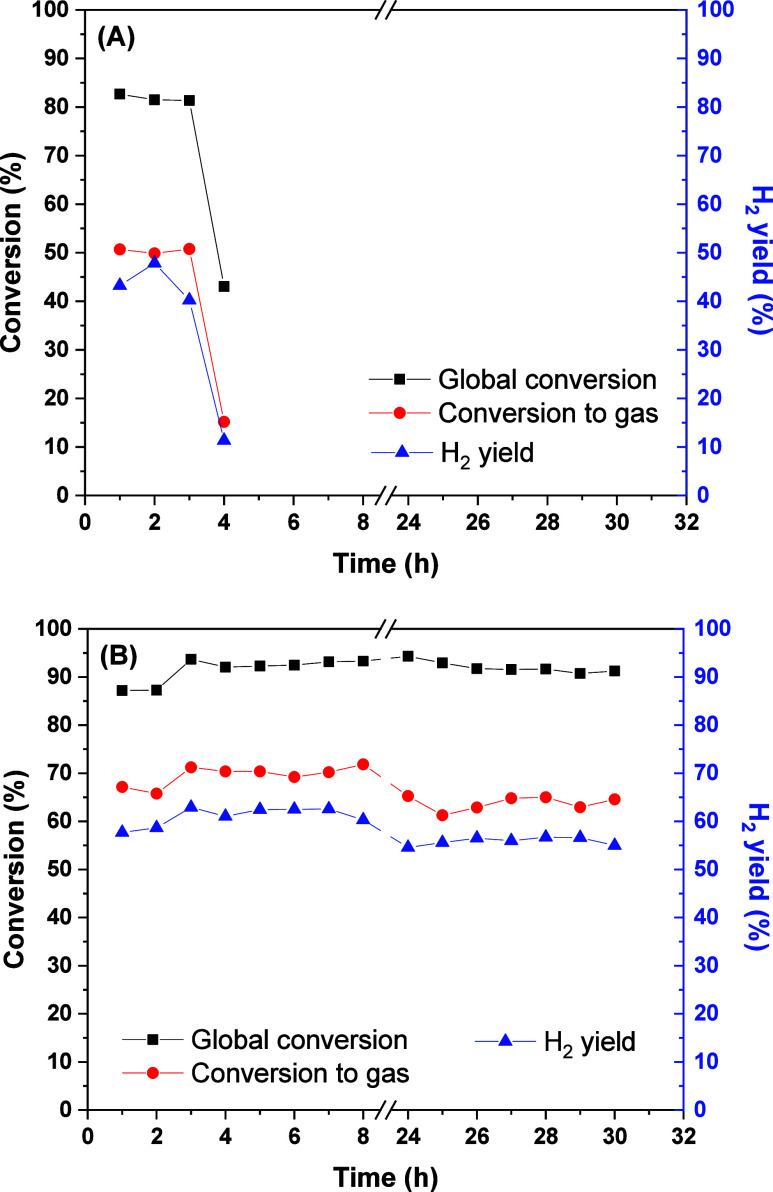
Stability tests for the (A) NiAl and (B) NiMgAl catalysts. Reaction conditions: 500 °C, 10% v/v ABE solution, GHSV = 50,000 h^–1^.

**Table 2:** Deactivation Degree, Coke Formation Rate Calculated from the Weight Loss in TGA Profiles and Global Conversions in the Stability Tests, and Ratio of the Intensity of the D/G Bands of Raman Spectra (I_D_/I_G_)

Catalyst	Deactivation degree (% h^–1^)	Coke formation rate (mmol_coke_ g_cat_^–1^ h^–1^ mol C_converted_^–1^)	I_D_/I_G_
NiAl	9.7	516	1.40
NiMgAl	0.0	13	1.16

Several side reactions are proposed to account for the coke formation, such as methane decomposition (eq 15), CO disproportionation (Boudouard reaction) (eq 16), and hydrogenation of CO and CO_2_ (eqs 17 and 18).^12–14^ Among these reactions, methane decomposition is favored at high temperatures (>600 °C), while the others are favored at lower reaction temperatures (<400 °C).^13^

15 $${\mathrm{CH}}_{4}⇌C+2H_{2}$$

16 $$2\mathrm{CO}⇌{\mathrm{CO}}_{2}+C$$

17 $$\mathrm{CO}+H_{2}⇌C+H_{2}O$$

18 $${\mathrm{CO}}_{2}+2H_{2}⇌C+2H_{2}O$$

In the temperature range used in the present study, coke formation may also be associated with ethylene polymerization or decomposition and acetone condensation, followed by oligomerization.^12,13,27,28^ Ethylene formation was observed only on the NiAl catalyst (Figure 2), which may explain its fast deactivation. However, when this catalyst was tested in ethanol steam reforming (results not shown here), no deactivation was observed at 500 °C, although there was significant ethylene formation. Thus, the deactivation in ABE reforming is mainly related to the presence of acetone, which can undergo oligomerization reactions, leading to numerous higher condensation products that form carbon deposits on the catalyst surface.^19,29^ The amount of coke and oligomers produced from acetone decreases by MgO addition, in accordance with Navarro et al.^19^

### 3.2 Characterization after the Stability Tests

The catalysts used in the stability tests were analyzed by XRD (Figure 4) to identify any modification in the crystalline structure compared to that of the reduced and calcined catalysts. The reduced catalysts presented peaks related to metallic nickel at 2θ equal to 44.5, 51.8, and 76.5° (JCPDS 04-0850).^21^ Ni peaks were maintained in the spent catalyst profiles, and no NiO peaks were detected, indicating the good stability of the metallic phase during the reaction. Moreover, a broad peak at 26° was observed in the spent catalysts related to coke deposition during the reaction.

**Figure 4:**
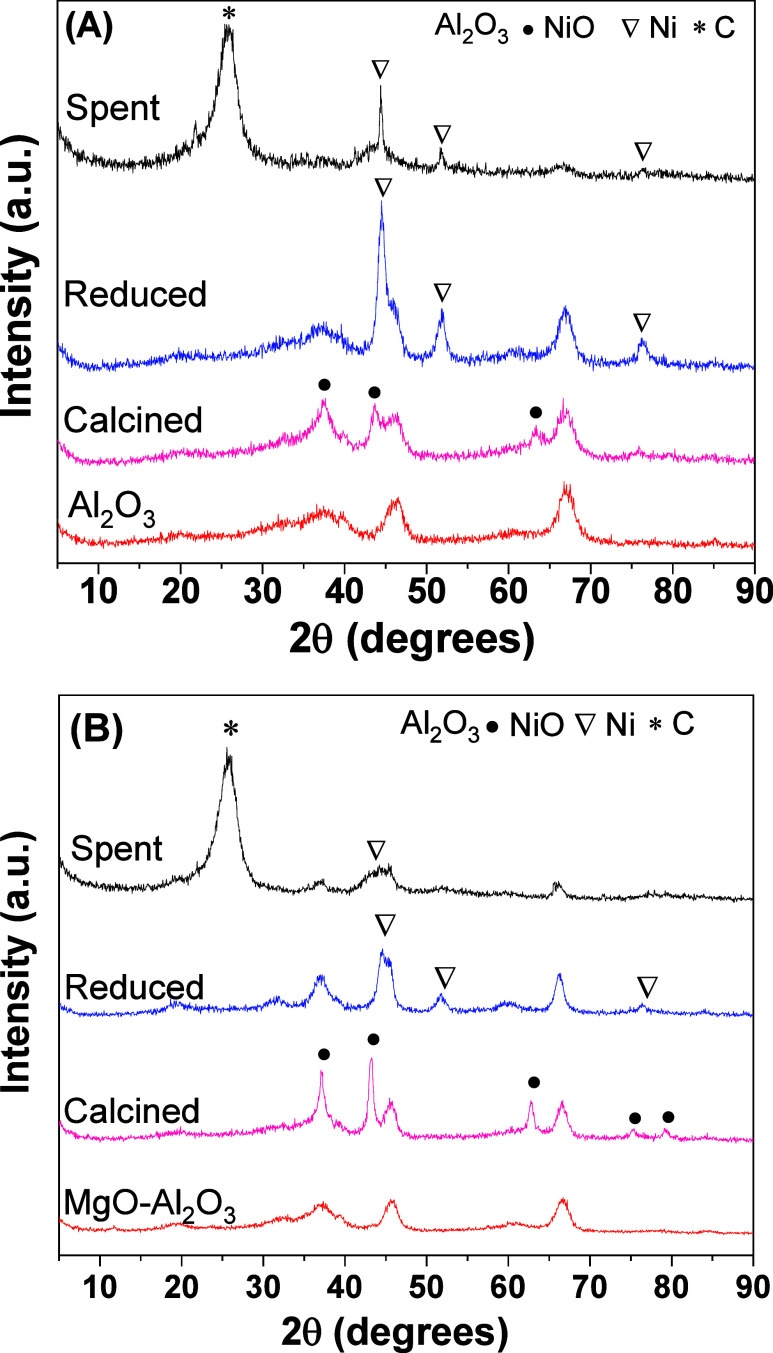
XRD patterns of the (A) NiAl and (B) NiMgAl catalysts after calcination, reduction, and use in the stability tests.

Figure 5 shows the TGA and DTA profiles of the catalysts used in the stability tests. These analyses were performed to calculate the amount of carbonaceous residues formed during ABE reforming and to evaluate the nature of the coke. The catalysts presented weight loss above 400 °C and exothermic peaks in the DTA profiles associated with the oxidation of deposited carbon species. The weight loss was similar for both catalysts (81% for NiAl and 88% for NiMgAl), but the reaction lasted only 4 h for NiAl, against 30 h for NiMgAl.

**Figure 5:**
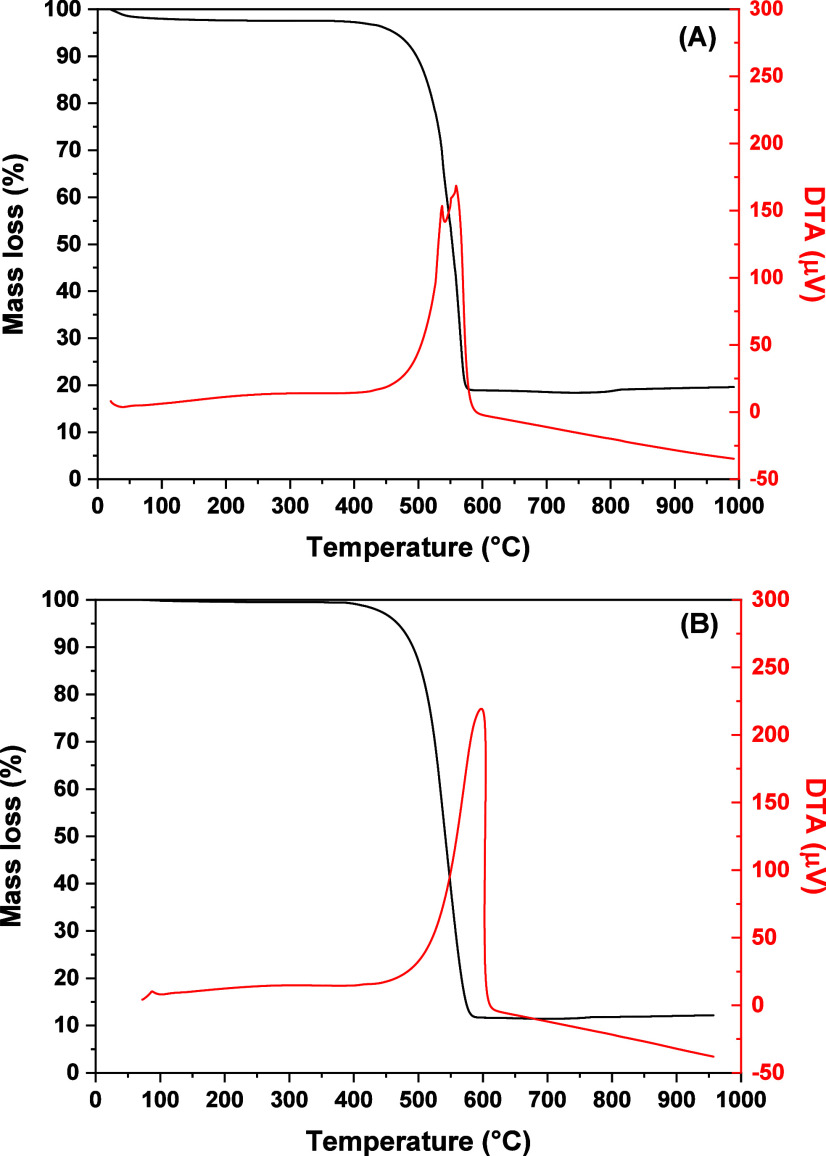
Thermogravimetric analysis (TGA) and differential thermal analysis (DTA) of the (A) NiAl and (B) NiMgAl catalysts after the stability test.

From the DTA profiles, the oxidation peak is divided into two maxima at about 537 and 560 °C for the NiAl catalyst, while there is a single peak at 595 °C for the NiMgAl. According to the literature, amorphous coke exhibits a lower oxidation peak compared to graphitic or filamentous coke.^30–32^ Graphitic or filamentous coke is generally oxidized at temperatures above 500 °C.^18,30,33^ Thus, the NiAl catalyst presented a more amorphous coke, although the formation of filamentous coke cannot be disregarded based on the temperature of the oxidation peaks shown in the DTA profiles. Amorphous coke is more harmful to the catalyst because it covers the active phase, encapsulating the metallic particles, and rapidly leads to deactivation.^34,35^ However, it is well-known that excessive growth of carbon filaments can cause reactor blockage,^14,36^ as observed for the NiAl catalyst. Vicente et al.^35^ suggested that ethylene and acetone are the encapsulating coke precursors, while CO (by means of the Boudouard reaction) and CH_4_ (to a lesser extent, by decomposition) were identified as the precursors of the filamentous coke. As ethylene was observed only for the NiAl catalyst (Figure 2), the formation of a more amorphous coke on this catalyst is in accordance with Vicente et al.^35^

The coke formation rate was measured as millimoles of carbon per gram of catalyst per hour per mole of carbon converted (mmol_coke_ g_cat_^–1^ h^–1^ mol C_converted_^–1^) and is presented in Table 2. The coke formation rate of NiMgAl was much smaller than that of NiAl, indicating its anticoking resistance. Adding MgO to the catalyst largely decreased the deposited carbon content and shifted the DTA peak to higher temperatures, showing that the basic sites are fundamental for reducing coke formation and leading to a less amorphous coke. The effect of MgO on decreasing coke formation in Ni/Al_2_O_3_ catalysts has already been reported in the literature for ethanol,^18,37^ acetone,^19,20^ and butanol^21^ reforming. The basic sites of MgO enhance water activation, which increases the available surface oxygen species to promote oxidation reactions of carbon precursors, thereby inhibiting carbon deposition on the catalyst surface.^18,21–24^ Furthermore, MgO promotes Ni dispersion due to a stronger interaction of Ni with the support and intercalation of the promoter between nickel and alumina.^18,21^ The increase in Ni dispersion is also crucial in inhibiting coke formation.

The graphitization order of the carbon deposited in the spent catalysts was evaluated by Raman spectroscopy (Figure 6). The catalysts presented two vibrational bands at 1324 cm^–1^ (D band) and 1597 cm^–1^ (G band). The D band is associated with the disorder-induced vibration of C–C bonds, such as in amorphous carbon. At the same time, the G band is related to the C–C vibration of well-ordered carbon material with an sp^2^ orbital structure, such as in a graphite layer.^31,32,38^ From Figure 6, it is observed that the D band is more intense than the G band, showing a greater formation of amorphous carbon. The ratio between the D and G bands (I_D_/I_G_) intensities was calculated, and the values are shown in Table 2. The catalysts presented a ratio of >1, indicating a predominance of amorphous carbon over graphitic carbon. The NiAl catalyst presented a higher I_D_/I_G_ ratio value, showing more amorphous carbon, in accordance with the DTA results. SEM images of the spent catalysts were used to evaluate the coke morphology (Figure 7). The deposition of amorphous spongy-type coke was mainly observed, corroborating the Raman results.

**Figure 6:**
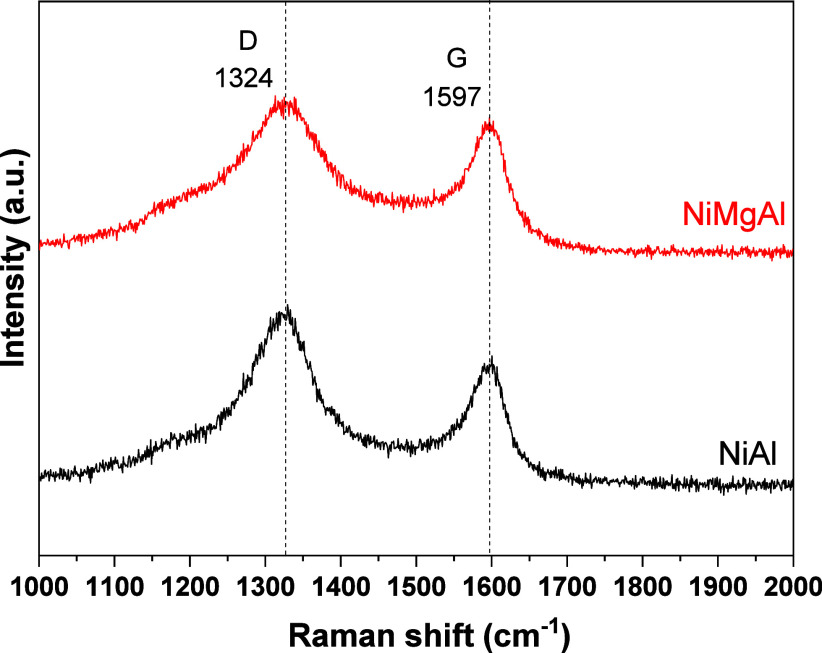
Raman spectra of the spent catalysts.

**Figure 7:**
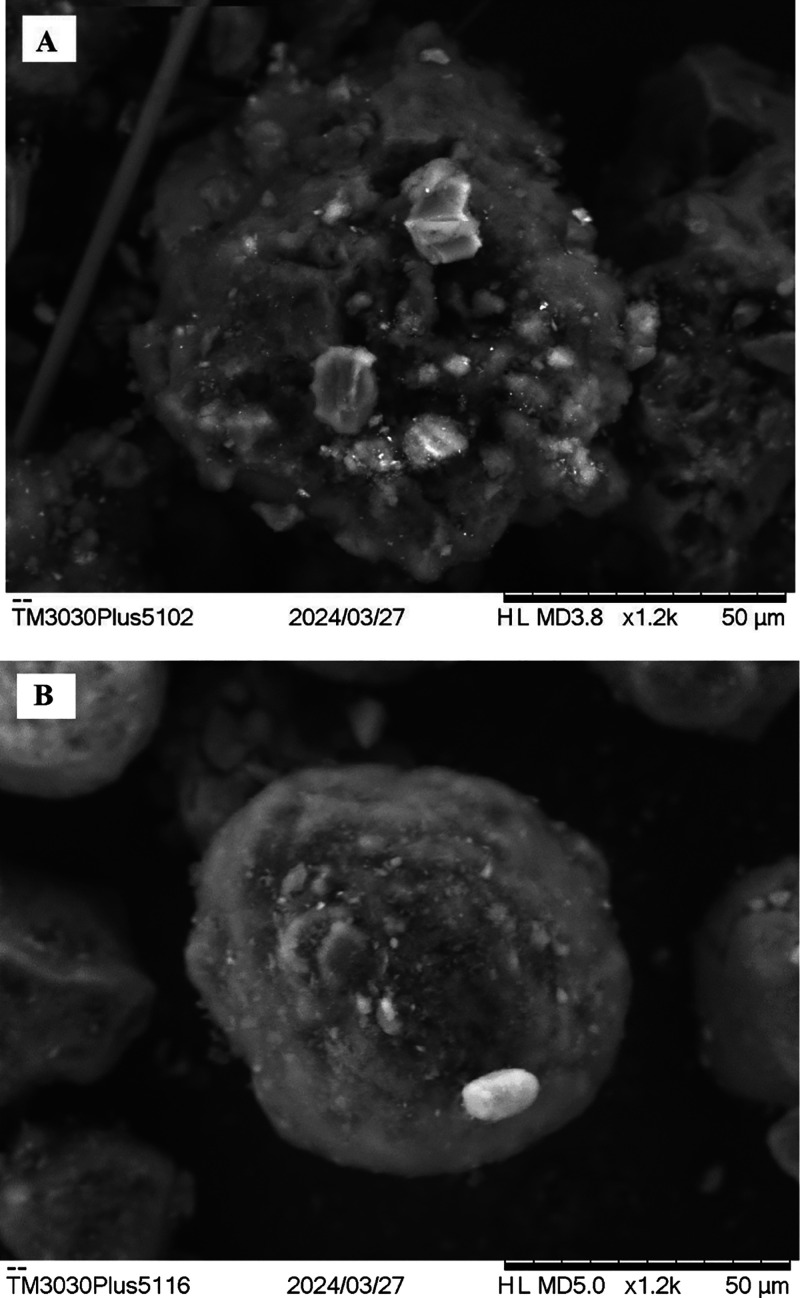
SEM images of the spent catalysts: (A) NiAl and (B) NiMgAl.

## 4 Conclusions

The present study introduces an alternative approach to producing hydrogen from renewable biomass feedstock. It demonstrates that the Ni/MgO-Al_2_O_3_ catalyst is a good choice for hydrogen production by steam reforming of the ABE (acetone–butanol–ethanol) mixture obtained from the fermentation of biomass-based substrates. The presence of MgO affects the catalyst properties, their activity and stability for ABE reforming, and the resistance to coke deposition. The promoted catalyst exhibited the highest Ni dispersion and basicity, with higher activity in ABE reforming and greater stability during 30 h at 500 °C. The Ni/Al_2_O_3_ catalyst exhibited similar activity as the promoted catalyst at the beginning of the reaction at 500 °C, but the reactor became entirely blocked after 4 h on stream. This catalyst showed the highest coke formation rate and more amorphous coke. Therefore, incorporating MgO into the catalyst is essential for its stability, significantly reducing carbon deposition.
